# Congenital Unilateral Agenesis of the Parotid Gland: A Case Report and Review of the Literature

**DOI:** 10.1155/2016/2672496

**Published:** 2016-11-08

**Authors:** Afshin Teymoortash, Stephan Hoch

**Affiliations:** Department of Otolaryngology, Head and Neck Surgery, Philipp University, Marburg, Germany

## Abstract

Congenital unilateral agenesis of the parotid gland is a rare condition with only few cases reported in the literature. A review of 21 cases in the available literature is presented in this article. We report on a further case of a 34-year-old woman with agenesis of the left parotid gland and lipoma of the right cheek. Clinicopathological characteristics of described cases in the literature were discussed.

## 1. Introduction

The major salivary glands start to develop between the sixth and seventh week of gestation beginning with the parotid gland which arises from ectodermal lining of the stomatodeum [1]. The submandibular and sublingual glands develop later and arise from the endodermal layer of the floor of the stomatodeum. Congenital absence of major salivary glands is a rare condition of unclear etiology. It is usually bilateral and sometimes associated with other development anomalies of the head and neck area. Unilateral agenesis of the parotid gland, especially, is an extremely rare condition with only few cases reported in the literature. The first report of a salivary gland agenesis was mentioned in 1885 by Gruber [2]. Since then, few cases of the unilateral submandibular gland agenesis have been reported in the literature [3].

Agenesis of parotid glands may occur alone or in association with anomalies of the submandibular or lacrimal gland, first brachial arch developmental disturbances, or other congenital anomalies [4–7]. The true incidence of agenesis of the parotid gland is difficult to ascertain because the condition is often asymptomatic [8]. Because saliva is mostly produced by other major and minor salivary glands, xerostomia does not occur and the absence of parotid gland is not noticed by the patient in the majority of cases [4].

We present a case of unilateral agenesis of the parotid gland in combination with a lipoma of the cheek on the opposite site. The clinical and radiological findings in this patient are described. A review of the unilateral parotid gland agenesis in the literature is also presented considering a summary of the data regarding gender, age, defect site, and combined manifestations.

## 2. Case Report

**Figure 1:**
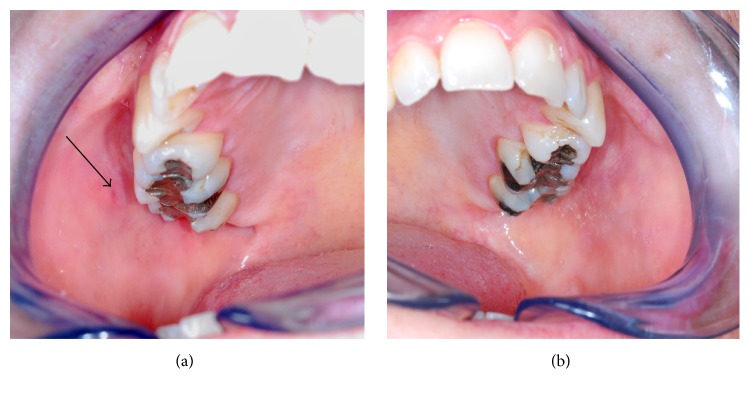
(a) Intraoral view of the right buccal mucosa shows the papilla of Stensen's duct. (b) Intraoral view of the left buccal mucosa. The papilla of Stensen's duct is absent.

A 34-year-old woman was referred to our department for evaluation of painless swelling of the right cheek over the last seven months. In addition, she often bit her right cheek. The swelling did not vary in size during eating and the patient had no other clinical symptoms and no history of recurrent parotitis. Xerostomia was not noted. There was no other relevant medical history and no family history of similar problems was reported. On clinical examination the oral mucosa was moistened by saliva. Bilateral hemifacial contour was normal, and there were no depressions in either preauricular region. Physical examination of the head and neck was without pathological findings, except for the absence of the left parotid gland papilla (Figure 1).

**Figure 2:**
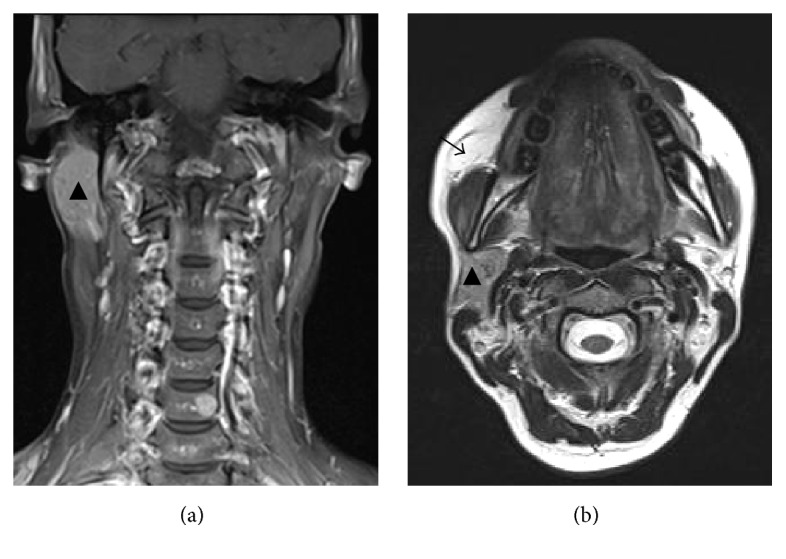
MRI scan of the parotid gland shows the unilateral agenesis of the parotid gland. (a) Coronary scan and (b) axial scan. The arrow points to the lipoma of the cheek and the triangle points to the right parotid gland.

**Figure 3:**
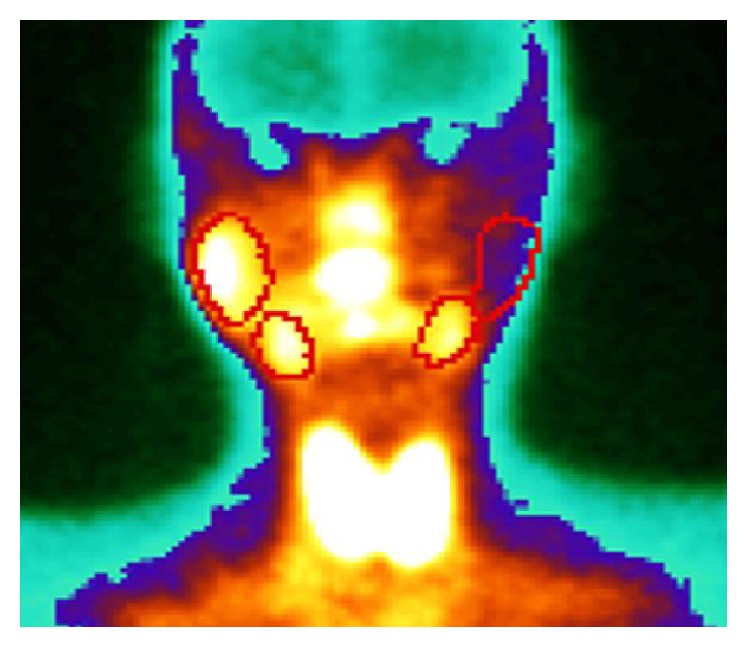
Technetium-99m pertechnetate scintiscan showing no activity in the left parotid gland and a normal activity in the other major salivary glands.

**Figure 4:**
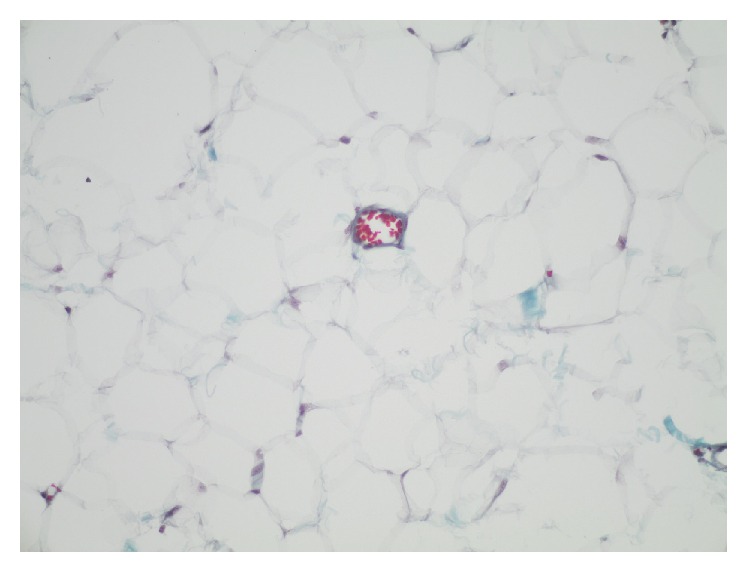
Histological examination of the buccal lipoma on the right side by Goldner's Trichrome staining.

Ultrasonographic examination of the head and neck area showed that the parotid gland on the left side was totally absent. The other major salivary glands were present without any pathology. A tumor in the right cheek ventral to parotid gland was observed with characteristic sonographic appearance of lipoma. For further evaluation of the tumor in the right cheek and assessment of the function of the other salivary glands magnetic resonance imaging (MRI) and scintigraphy with Technetium (Tc-99m) sodium pertechnetate were performed. MRI confirmed a lipoma of the cheek on the right side and a unilateral absence of the left parotid gland (Figure 2). Other pathological findings in the head and neck area could not be found. Salivary gland scintigraphy showed no activity in the area of the left parotid gland with normal function of the other major salivary glands (Figure 3). The patient had no clinical symptoms associated with the absence of the parotid gland. The buccal tumor was removed via parotidectomy incision and exposition of the facial nerve. Histological examination of the specimen confirmed the clinical suspicion of lipoma (Figure 4). The postoperative recovery proceeded without complications. There was no further follow-up after wound healing was accomplished.

## 3. Discussion

Congenital absence of the salivary glands is a rare condition which has been described to affect the parotid or submandibular glands [26]. Agenesis of salivary glands may be unilateral or bilateral and multiple major salivary glands can be involved [27–29].

The true incidence of unilateral agenesis of the parotid gland is difficult to ascertain because it is often asymptomatic [10]. Congenital unilateral absence of the parotid gland is uncommon with only few cases reported. The absence of bilateral parotid glands has been observed in lacrimoauriculodentodigital (LADD) syndrome [30], in hypoplasia of the lacrimal glands or absence of lacrimal puncta [31], in hemifacial microstomia, and in ectodermal dysplasia. The resulting disturbances affect primarily the lacrimal glands, the inner and outer ear, the salivary glands, and the osseous frame work [24, 30]. Aplasia of the major salivary glands may be associated with aplasia/hypoplasia of the lacrimal glands. This condition is confirmed as autosomal dominant disorder [32]. Single cases of bilateral parotid gland agenesis associated with cleft lip and palate, Down syndrome, or Klinefelter syndrome have been reported [1, 33–35]. Some cases of familial salivary gland agenesis have also been documented [31]. Bilateral forms of agenesis could be responsible for a severe lack of saliva causing dental caries, periodontal disease, and candidosis [8].

In the available literature, only 22 cases of unilateral agenesis of the parotid gland have been described including the present case (Table 1). Among the 22 cases, 11 (50%) of the patients were male and 11 (50%) were female. At the time of diagnosis the youngest patient was 50 days old and the oldest was 75 years old with an average age of 34.7 years. The unilateral absence of the right parotid gland was nearly twice as frequent as the left side (14/8 cases). The papilla of Stensen's duct was present in only one case. In the other cases the parotid papilla was absent (*n* = 12) or the presence of the parotid papilla was not documented (*n* = 9).

In most reported cases the unilateral agenesis of the parotid gland was associated with a painless swelling of the contralateral parotid gland or facial asymmetry without any other significant clinical symptoms [9, 10, 13–15, 17, 20, 25]. According to the authors the swelling of the contralateral parotid gland was as a compensatory functional hypertrophy of the parotid gland [14, 15, 17, 25]. Association with other pathologies of the head and neck area could not be found in those cases. Sialosis of the contralateral parotid gland was found in one case; the diagnosis was confirmed by an open biopsy of the parotid gland [9]. One case showed ipsilateral agenesis of the parotid gland in association with first branchial cleft cysts [12]. Another two patients suffered from lateral facial cleft associated with accessory mandible [7, 24]. Pleomorphic adenoma of the contralateral parotid gland, the ipsilateral accessory parotid gland, or buccal space was reported in each case [18, 19, 23]. In another case agenesis of parotid gland was masqueraded in I-123 metaiodobenzylguanidine scan with SPECT/CT by a metastasis of a left craniocervical neuroblastoma [21]. In other cases agenesis of the parotid gland was associated with ipsilateral angioma of the cheek and ipsilateral accessory parotid tissue [8, 11, 16, 22]. The present case described a patient with agenesis of the left parotid gland and a lipoma of the right cheek.

The unilateral agenesis of the parotid gland may be clinically silent. Clinical suspicion should arise in cases of asymmetrical parotid areas and a painless unilateral swelling of the parotid gland. Clinical examination, especially the absence of the papilla of Stensen's duct, could be helpful for diagnosis. Mostly the unilateral agenesis of the parotid gland seems to be a coincident finding. We were able to confirm the diagnosis of parotid gland agenesis by using a combination of MRI and salivary gland scintigraphy.

## Figures and Tables

**Table 1:** Reported cases of unilateral parotid gland agenesis in the literature (*n* = 22).

Number	Authors	Year	Age	Sex	Site	Combined manifestations	Papilla of Stensen's duct
1	Kelly et al. [9]	1990	28	m	Right	Sialosis of contralateral parotid gland	Absent
2	Almadori et al. [10]	1997	38	m	Left	Hypertrophy of contralateral parotid gland	Absent
3	Bhide and Warshawsky [11]	1998	16	m	Right	Ipsilateral accessory of parotid tissue	Unknown
4	Sichel et al. [12]	1998	4.5	f	Right	First branchial cleft cyst type II	Unknown
5	Hyang et al. [13]	1999	22	f	Left	Hypertrophy of contralateral parotid gland	Unknown
6	Martínez Subías et al. [14]	2000	21	f	Right	Hypertrophy of contralateral parotid gland	Unknown
7	Daniel et al. [15]	2003	5	m	Right	Hypertrophy of contralateral parotid gland	Unknown
8	Salvinelli et al. [16]	2004	53	m	Right	Ipsilateral angioma of the cheek	Absent
9	Martín-Granizo and García-González [17]	2004	58	m	Right	Hypertrophy of contralateral parotid gland	Absent
10	Karakoc et al. [18]	2005	35	f	Left	Pleomorphic adenoma of contralateral parotid gland	Absent
11	D'Ascanio et al. [8]	2006	53	f	Right	Hypoplasia of the thyroid's right lobe and homolateral angioma of the cheek	Absent
12	Lee [19]	2010	65	f	Right	Pleomorphic adenoma in the ipsilateral buccal space	Unknown
13	Chen et al. [20]	2011	75	m	Right	Contralateral compensation hypermetabolism of FDG	Unknown
14	Udall and Cho [21]	2011	0.8	m	Right	Metastases from left craniocervical neuroblastoma	Unknown
15	Capaccio et al. [22]	2012	44	m	Right	Recurrent inflammation of accessory parotid tissue	Present
16	Seith et al. [23]	2013	41	m	Left	Pleomorphic adenoma of ipsilateral accessory parotid gland	Absent
17	Sun et al. [24]	2013	15	w	Left	Partial duplication of the mandible facial cleft, accessory parotid gland	Unknown
18	Günbey et al. [25]	2014	45	w	Right	Hypertrophy of contralateral parotid gland	Absent
19			52	w	Left		Absent
20			63	m	Left		Absent
21	Özçelik et al. [7]	2014	0.1	w	Right	Ipsilateral facial cleft, accessory mandible, facial weakness	Absent
22	Present case	2016	30	w	Left	Contralateral cheek lipoma	Absent

m = masculine; f = feminine; FDG = fluorodeoxyglucose.
